# In pursuit of a better transition to selected residencies: a quasi-experimental evaluation of a final year of medical school dedicated to the acute care domain

**DOI:** 10.1186/s12909-022-03871-0

**Published:** 2022-11-23

**Authors:** Gersten Jonker, Eveline Booij, Jacqueline E. M. Vernooij, Cor J. Kalkman, Olle ten Cate, Reinier G. Hoff

**Affiliations:** 1 Department of Anesthesiology, University Medical Center Utrecht, Utrecht University, PO Box 85500, 3508 GA Utrecht, the Netherlands; 2 Simulation Center, Rijnstate Hospital, Velp, the Netherlands; 3 Center for Research and Development of Education, University Medical Center Utrecht, Utrecht University, Utrecht, the Netherlands

**Keywords:** Undergraduate medical education, Transition Postgraduate training, Specialty orientation, Preparedness, Competence, Assessment, OSCE, Simulation, Pre-test post-test design, Acute care

## Abstract

**Background:**

Medical schools seek the best curricular designs for the transition to postgraduate education, such as the Dutch elective-based final, ‘transitional’ year. Most Dutch graduates work a mean of three years as a physician-not-in-training (PNIT) before entering residency training. To ease the transition to selected specialties and to decrease the duration of the PNIT period, UMC Utrecht introduced an optional, thematic variant of the usual transitional year, that enables the development of theme-specific competencies, in addition to physicians’ general competencies.

**Methods:**

We introduced an optional transitional year for interested students around the theme of acute care, called the Acute Care Transitional Year (ACTY). This study aimed to evaluate the ACTY by judging whether graduates meet postgraduate acute care expectations, indicating enhanced learning and preparation for practice.

In a comprehensive assessment of acute care knowledge, clinical reasoning, skills, and performance in simulations, we collected data from ACTY students, non-ACTY students interested in acute care, and PNITs with approximately six months of acute care experience.

**Results:**

ACTY graduates outperformed non-ACTY graduates on skills and simulations, and had higher odds of coming up to the expectations faculty have of a PNIT, as determined by global ratings. PNITs did better on simulations than ACTY graduates.

**Discussion:**

ACTY graduates show better resemblance to PNITs than non-ACTY graduates, suggesting better preparation for postgraduate acute care challenges.

**Conclusion:**

Transitional years, offering multidisciplinary perspectives on a certain theme, can enhance learning and preparedness for entering residency.

**Supplementary Information:**

The online version contains supplementary material available at 10.1186/s12909-022-03871-0.

## Background

Transitions within the medical education continuum have enjoyed the attention of medical educators for a long time [1, 2]. Learners experience difficulties in adapting to the expectations of the new phase when moving from classroom education to clinical clerkships [3], from medical school to residency [4, 5], and from postgraduate training to fellowship or practice [6–8]. Overall, as learners advance to the next phase, they frequently experience an incomplete set of competencies for that phase. In particular, the undergraduate to postgraduate transition has received attention and schools and countries have dealt with this transition in different ways [9, 10]. The UK has introduced a two-year Foundation period, whereas the USA and Canada do not apply any transitional phase but require all graduates to start residency with an internship year, and many other countries require one or more years of clinical service experience [11].

Medical schools struggle to seek the best curricular structures to ease the transition to residency. Introducing longitudinal, integrated clinical experiences [12] and creation of an education continuum within a single specialty [13] are examples.

In the Netherlands, most graduates start their careers working as a physician-not-in-training (PNIT), assuming clinical responsibilities and expanding clinical experience before entering residency [14–16]. As a licensed doctor, a PNIT works in hospital patient care with clinical oversight of a medical specialist and with varying levels of supervision, provided by a resident or specialist. This voluntary phase of practice without formal education [14], is of variable duration – from less than one to over three years – and originated from a surplus of graduates for residency positions. On average, graduates work 34 months as a PNIT before starting postgraduate training [17]. Residency selection procedures happen in an open market model, and committees have become accustomed to experienced candidates. This competitive process has urged applicants to expand their clinical experiences, and even adding doctoral research degrees, to maximize their chances to be selected for highly sought residencies. Dutch medical schools, as well as the government, have become aware of the need to better align medical school and residency, to decrease the total length of preparatory training and clinical practice before residency, and thus shorten the whole trajectory of specialization. Dutch medical school reforms in the 2000 decade have led to an elective-based final, ‘transitional’ year model [14, 18, 19]. The primary goal of the transitional year is to improve graduates’ readiness for work by learning to carry clinical responsibilities, and in addition it potentially reduces the duration of the PNIT period by decreasing the need or time of having to learn carrying responsibilities postgraduately. This final, transitional year contains substantial elective opportunities to help students grow toward the responsibilities of a doctor under appropriate supervision, obtain clinical experience and expertise, explore career options, and improve chances on the job market [19, 20]. At University Medical Center (UMC) Utrecht, final year students assemble an individual program [14, 19]. Students spend a 12 weeks sub-internship in a broad clinical specialty of their choice in which they take responsibility for a limited number of "own" patients. They expend another 6 to 12 weeks to one or two clinical electives at a sub-internship level. In addition, students do 12 to 18 weeks research in a domain of choice. They use another six weeks for mandatory training courses in research, teaching and professionalism.

Instead of composing a fully individual package of rotations, students may opt for a thematic track during their transitional year that enables the development of specific competencies within a medical domain (the theme), in addition to the general competencies that graduates need. A thematic track offers focused preparation and professional orientation within the domain of medicine.

### Aim of the study

In pursuit of a better transition to work in selected residencies and specialties, the current study aimed to evaluate to which degree a thematic final year track, geared to specialties in a limited domain, could prepare students better for clinical work as a doctor (either as a PNIT or entering residency). We combined a scholarly approach to curriculum design and assessment with the collection of research data, and chose the domain of acute care.

## Methods

### Setting

In 2015, we introduced an optional Acute Care Transitional Year (ACTY) at the UMC Utrecht to ease the transition to not just any postgraduate training or non-training post, but to a selected set of residencies or specialties. Per year, on average 18 students interested in acute care (about 7% of an annual cohort) enroll in the ACTY [21].

Anesthesiology, cardiology, emergency medicine, intensive care medicine, and pulmonary medicine have a shared interest in acute care and faculty members of these five specialties designed and implemented the ACTY collaboratively. The three goals of the ACTY are: (a) to enhance acute care learning by boosting motivation, (b) to prepare graduates for clinical acute care practice, and (c) to offer a focused specialty orientation.

The ACTY strives to enhance learning through a coherent final year program that brings together a group of students who share a profound interest in acute care. ACTY’s learning objectives are the acute care challenges recently graduated doctors in several specialties may face. The tasks, formulated as entrustable professional activities (EPAs), link the learning objectives directly to postgraduate expectations [22, 23]. The educational team created three ACTY EPAs: ‘Recognition and initial treatment of patients with vital instability’, ‘Evaluation of patients with respiratory insufficiency’, and ‘Evaluation of patients with circulatory insufficiency’ (See Appendix 1 for full descriptions).

In monthly three-hour teaching sessions, all ACTY students and teachers discuss acute care content related to the EPAs. We designed these sessions to foster a learning community of highly motivated, congenial peers [24]. The sessions present a forum for teacher-guided peer reflections on clinical situations students experienced. The educational sessions do not include skills training or resuscitation drills.

Students commencing the ACTY take an extensive multimodal formative pre-test blueprinted to the ACTY EPAs, to assess knowledge, clinical reasoning, skills, and performance in high-fidelity simulations. The test is designed as a motivating and eye-opening starter to set the stage for the final year, to identify both existing strengths and areas for improvement, and to guide personal learning during the subsequent clinical rotations [24, 25].

ACTY students choose three out of five participating specialties for one sub-internship of twelve weeks and two of six weeks. Additionally, students take a twelve-week research elective in one of the five ACTY specialties.

At completion of the ACTY, students take a formative post-test, similar to the pre-test, to identify the degree of personal growth, provide insight in acute care competence, and detect areas for further learning. The test is mandatory, but not created to serve as a pass/fail assessment.

The ACTY provides orientation on a career within the acute care domain. The ACTY targets the sweet spot between breadth and depth in a final year. Breadth is essential to deliver broadly trained physicians; depth is vital for individualization and pre-specialization. The track offers concentrated but balanced clinical experience, without being overly focused or narrowed down to a single specialty [26]. The ACTY aims to equip graduates with acute care competence, useful for doctors aspiring a career in the ACTY specialties or in other fields involved in acute care (e.g. general practice, tropical medicine, or surgery). Simultaneously, the year allows students to discover which specialty within the acute care domain best matches their interests, while educators can gauge the fit of a student within the specialty. Specialty orientation is a worthwhile goal. One third of students make their choice in the final year and the majority indicate that final year experiences have a strong influence on their ultimate choice of specialty [27]. All ACTY students are assigned to a senior postgraduate trainee in one of the five acute care specialties. This trainee acts as a buddy, provides ‘mentoring on demand’, supports the acculturation to the specialty, gives career advice, and helps to validate the student's choice for this direction.

We have reported before on early experiences with the program [24] and on experiences with the simulations [25]. The current study aims to evaluate to which degree ACTY’s graduates meet postgraduate expectations of junior doctors to provide acute care, as an indication of enhanced learning and preparation for clinical practice.

### Design

We collected pre-test and post-test data from ACTY students and participants in two comparison groups in a quasi-experimental design.

### Recruitment

All students who commenced the ACTY from September 2015 onwards (small cohorts could start every six weeks) were eligible to participate in the study. We aimed to obtain post-test results from 25 ACTY participants.

To value ACTY test results we included two comparison groups. The first group consisted of UMC Utrecht students who did not take part in the ACTY track and composed an individual transitional year program. To match these participants for motivation and affinity with acute care, we invited students who had chosen at least one sub-internship in anesthesiology, cardiology, emergency medicine, intensive care medicine, or pulmonology for participation in the pre-test and post-test by email. Despite sending reminders, we experienced difficulty in recruiting student controls at the start of the transitional year. We figured that graduating students might be interested to obtain insight in their level of acute care competence and late 2017 we decided to recruit similarly matched non-ACTY medical students around graduation for a single post-test comparison.

A second comparison group contained recently graduated, junior doctors currently employed as physicians, not yet in a residency (PNITs). We recruited PNITs with three to nine months of clinical experience in ACTY specialties, by asking regional hospital departments and educationalists to distribute recruitment emails and reminders. Participating PNITs had not graduated from the ACTY.

### Procedure

Participants took part in the comprehensive multimodal assessment of acute care knowledge, clinical reasoning, clinical skills, and performance in high-fidelity simulated situations. Table 1 provides an overview of the assessment modalities (further details are provided in Appendix 2). The ACTY EPAs served as an assessment blueprint, i.e. as assessment elements we included the knowledge, skills, and attitudes listed in the EPA descriptions (Appendix 1).

**Table 1:** multimodal acute care assessment and data generated

Assessment modality	Test description	Examples of stations	Number of stations	Scores generated per participant	Analyzed at group level as …
**Written knowledge exam** • 40 minutes • around 40 closed and 6 open format questions	• paper-based • to assess factual and applied knowledge and higher order thinking		1	1 knowledge score	Mean knowledge score
**Case-based discussions (CBDs)** • 2x 10 minutes • patients requiring urgent attendance at the ED	• one-on-one structured questioning • to assess information gathering, clinical reasoning, and know-how of case management	A 65-year old male presenting with chest pain at the ED	2	2 CBD scores	Mean CBD score
		A 38-year old female presenting with dyspnea and sharp pain on breathing at the ED		2 GRS	GRS distribution
**Skills stations** • 5-7x 5 minutes	• OSCE format • to assess skill performance	Bag-mask ventilation	5-7	5 – 7 OSCE scores	Mean OSCE score
		Defibrillation		5 – 7 GRS	GRS distribution
		ECG interpretation			
**High-fidelity simulations** • 3x approximately 12 minutes • challenges a PNIT could encounter at the ED or on the ward • participant enacted the role of first responding doctor. A non-obstructive nurse was present, who only acted on instruction.	• real-time scenarios with standardized scripts • to assess clinical reasoning, clinical judgment, technical skills, and behavioral skills and attitudes, including communication and crisis resource management • certified simulation facilitators • debriefing after each scenario to foster learning and well-being	A 60-year old male is reported to be “just not right” in the urology ward	3	3 simulation scores	Mean simulation score
		A 21-year old comatose male is brought in from a party to the ED by paramedics		3 GRS	GRS distribution

*CBD* Case-Based Discussion, *GRS* Global rating score, *OSCE* Objective Structured Clinical Examination, *ED* Emergency Department, *ECG* Electrocardiogram, *PNIT* Physician-not-in-training

### Assessors

Each CBD and assessor-observed OSCE station was supervised and assessed by an individual clinician with appropriate expertise who volunteered to assess several students during the day. One of the researchers (GJ) assigned clinicians (residents or specialists) to stations that matched their experience and qualifications. One OSCE station was unmanned and written choices made by the participant were judged afterwards by researchers GJ and EB. The high fidelity simulation assessments were scored by joint effort of EUSim certified simulation center staff. Altogether, during a complete pre- or post-test a participant would be observed and scored by eight to twelve qualified clinical assessors. Inter-rater variability and rater bias were mitigated by extensive individual briefings prior to the assessment done by researchers GJ and EB. This briefing comprised information on the rationale and use of the scoring rubric, test duration, and expected performance level.

Assessors evaluated all participants as if they were a PNIT with six months of clinical experience. Assessors were not made aware whether participants were from intervention or comparison groups, nor whether they did a pre-test or a post-test. Assessors scored participants’ performance live on a paper checklist.

### Assessment scores

Each station had a specific checklist following the chronology of the consultation, steps in skill execution, or expected actions and important steps in case management. Assessors scored each item on a three-point scale (done adequately – done incompletely or not timed well – done insufficiently or omitted).

Every assessment element (e.g. station) yielded a summary score, calculated as the awarded points as percentage of the maximum points. From the element summary scores a mean modality score could be calculated for CBDs, for OSCEs, and for simulations.

On CBDs, assessor-observed OSCEs, and simulations, assessors evaluated participant performance with a global rating score (GRS), in addition to checklist scores. This score was a holistic, gestalt, expert evaluation of overall performance on a three-point scale (does not meet expectations – borderline – meets expectations) in answer to the question “How did the participant’s performance compare to the expected level of a PNIT with six months of clinical experience?”. We chose the arbitrary level of six months to have assessors think of a graduate with substantial but delineated experience and to make a match with the actual experience of the PNIT comparison group. Table 1 summarizes the data generated by the assessment.

We ran 25 highly similar test sessions, consisting of two separate half-days. In one test session, all participants would take the same test, either as pre-test or as post-test. Participants’ pre-test and post-test content was never identical. Students attended the pre-test in the first month of their final year and the post-test near the end of the year. Recruited as recent graduates, PNITs could not take a pre-test at the start of their final year. Thus, PNITs attended the test once and, in the comparison, this is regarded as a post-test. All participants received feedback on their performance in the tests.

### Ethical approval

The ethical review board of the Netherlands Association for Medical Education approved the study (NERB file 369, November 2014). All participants gave written informed consent.

### Analysis

Modality scores (i.e. knowledge score, mean CBD score, mean OSCE score, and mean simulation score) are expressed in percent points and are compared as group means with SD and differences with 95% CI in rounded percent points.

We explored differences between groups in mean modality scores, in the pre-test, the post-test, and in gain between pre- and post-test, for statistical significance with one-way ANOVA. We used post hoc independent samples t-tests to evaluate differences between two groups.

We used cumulative odds ordinal logistic regression with proportional odds to determine the effect of group type on domain GRS. We report differences between groups as odds ratios.

## Results

All 33 ACTY students during the inclusion period participated in the study (group ACTY). They spent 24 weeks in three different sub-internships in anesthesiology (24.2% of rotations chosen), cardiology (25.3%), emergency medicine (18.2%), intensive care medicine (27.3%) and pulmonology (5.1%).

The 21 participants in the student comparison group (group C), 10 for the pre-test and post-test, and 11 for the post-test only, had chosen a median of 2 acute care related sub-internships (range 1-3), spending a median of 12 weeks (range 6-24) in anesthesiology (15% of rotations chosen), cardiology (25%), emergency medicine (20%), intensive care medicine (20%) and pulmonology (15%).

The 17 PNITs (group P) had a mean clinical experience of 5.5 months (range 3.5 – 8.0) in anesthesiology (6%), cardiology (12%), emergency medicine (24%), or intensive care medicine (59%).

**Fig. 1:**
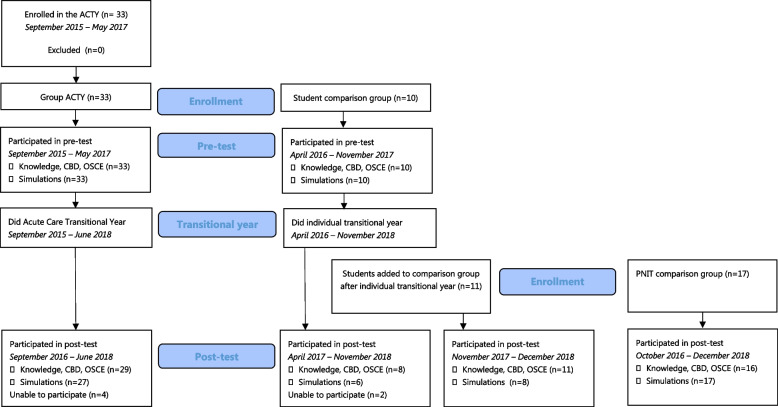
Participant flow in pre- and post-test

Figure 1 participant flow. No participants withdrew from the study. Some participants in group ACTY and group C were unable to attend the post-test. In all groups, a few participants could only partly attend the post-test.

### Test modality scores

At baseline, there were no statistically significant differences between group ACTY and group C in mean scores on the knowledge test, CBDs, and OSCEs (for statistical details, see Appendix 3). On simulations, there was a statistically significant difference in means, with students in group ACTY having significantly higher scores than students in group C (*p*=.026, mean difference 6 (2), 95% CI 1 – 11).

In the post-test (Table 2), there were no statistically significant differences in group means on the knowledge exam and CBDs (Appendix 3). There were statistically significant differences in group means for OSCEs (*p*=.01) and simulations (*p*=.001). Group ACTY (*p*=.004, mean difference 8 (3), 95% CI 3 – 13) and group P (*p*=.026, mean difference 5 (2), 95% CI 1 – 10) had significant higher mean scores on OSCEs than group C.

**Table 2:** Post-test mean scores (in percentage of maximum score) on four assessment modalities

Assessment modality	Group ACTY			Group C			Group P		
	n	Mean (SD)	95% CI	n	Mean (SD)	95% CI	n	Mean (SD)	95% CI
Knowledge	29	67 (11)	63 – 71	19	63 (13)	56 – 69	16	67 (7)	63 – 70
CBDs	29	79 (11)	75 – 84	19	72 (10)	67 – 77	16	79 (9)	74 – 84
OSCEs	29	74 (9) *	71 – 78	19	67 (8) *†	63 – 70	16	72 (5) †	69 – 75
Simulations	27	65 (8) ‡ **	62 – 69	14	59 (11) ‡§	52 – 65	17	71 (6) § **	68 – 74

*ACTY* Acute Transitional Year group, *C* Student comparison group, *P* PNIT comparison group. *CBD* Case-based discussion, *OSCE* Objective structured clinical examination. Assessment modality scores expressed as % of maximum obtainable score, as group mean (SD= standard deviation) and 95% confidence interval (CI). * *p*=.004; † *p*=.026; ‡ *p*=.036; § *p*<.001; ** *p*=.019

Group ACTY (*p*=.036, mean difference 7 (3), 95% CI 1 – 13) and group P (*p*<.001, mean difference 12 (3), 95% CI 6 – 18) had significantly higher mean scores on simulations than group C. Group P had a significantly higher mean score on simulations than group ACTY (*p*=.019, mean difference 6 (2), 95% CI 1 – 10).

There were no statistically significant differences between means in gain between pre-test and post-test scores of group ACTY and group C for all assessment modalities (Appendix 3).

### Global rating scores

In all groups in the post-test, on global ratings of overall station performance, assessors most often scored “meets expectations” on CBDs and least often on simulations (Table 3).

**Table 3:** Global rating scores (GRS) in post-test per assessment modality

	Group ACTY n (%)	Group C n (%)	Group P n (%)
**GRS on CBDs**			
Does not meet expectations	2 (3.4)	4 (11.1)	0 (0)
Borderline	7 (12.1)	7 (19.4)	2 (6.5)
Meets expectations	49 (84.5)	25 (69.4)	29 (93.5)
Pie chart (%)			
**GRS on OSCEs**			
Does not meet expectations	12 (7.4)	12 (13.2)	4 (4.9)
Borderline	25 (15.3)	27 (29.7)	15 (18.5)
Meets expectations	126 (77.3)	52 (57.1)	62 (76.5)
Pie chart (%)			
**GRS on Simulations**			
Does not meet expectations	18 (22.5)	14 (33.3)	4 (7.8)
Borderline	25 (31.3)	17 (40.5)	10 (19.6)
Meets expectations	37 (46.3)	11 (26.2)	37 (72.5)
Pie chart (%)			

Frequency of scores, per group type. Pie charts showing relative occurrence of global rating scores on CBDs, OSCEs, simulations

*ACTY* Acute Care Transitional Year, *C* Student comparison group, *P* PNIT comparison group. *CBD* Case-based discussion, *OSCE* Objective structured clinical examination. Because each participant did more than one station per modality, n refers the respective score frequency

ACTY students more frequently scored “meets expectations” than group C students. Ordinal logistic regression showed that group type had a statistically significant effect on the prediction of getting a high GRS on CBDs, OSCEs, and simulations (see Appendix 3 for a comprehensive report of the model). This finding can be rephrased with odds ratios of getting a high GRS, using group C as a reference (or: standard). The odds of participants in group ACTY getting a high GRS were 2.06 – 2.49 that in group C, statistically significant for OSCEs and simulations. Participants in group P had statistically significant higher odds (OR 2.45 – 6.69) of receiving a high GRS than those in group C. Also, the odds of obtaining a high GRS in group P was 2.70 for CBDs, 0.99 for OSCEs, and 3.23 for simulations compared to group ACTY; a statistically significant difference on simulations (Appendix 3).

## Discussion

We implemented a thematic transitional year focused on acute care (ACTY) and collected data in a multimodal assessment to evaluate whether ACTY graduates meet postgraduate expectations of junior doctors to provide acute care.

Students graduating from ACTY performed better in the assessment than non-ACTY students with an interest in acute care, especially on performance test modalities (i.e. skills and simulations). Moreover, ACTY students had higher odds than non-ACTY students of meeting the expectations of a PNIT, as determined by GRS. Thus, ACTY graduates show better resemblance to PNITs than non-ACTY graduates, suggesting they are better prepared for junior doctor acute care challenges.

However, PNITs with a mean of 5.5 months of clinical experience did better than students graduating from ACTY on simulations, both in scores and GRS. This suggests that PNITs have a steep learning curve in those first months of ‘real’ clinical work following graduation. Their clinical experience helped in showing an “organized, orderly approach” [28]. Furthermore, PNITs starting in a first acute care job often take additional acute care training. Certification as an advanced life support provider has a medium-size effect on performance in a simulation test of acute care competence [28]. Of interest, a few GRS awarded to PNITs were below assessor expectations of PNITs with six months of experience. This may indicate the recent graduates’ incomplete preparedness for acute care and imperfect or inhomogeneous competence development. However, also failure of an occasional test station in a standardized test may explain this finding.

In times of great need for labor force, such as currently (2020 – 2022) with the Covid-19 pandemic [29, 30], demonstrable competence, obtained as a student in the transitional year, is a way to select the right graduates for critical junior doctor positions. We postulate that ACTY graduates, performing approximately on a midpoint between non-ACTY graduates and acute care PNITs, have a head start for deployment in such jobs.

The ACTY is an example of a thematic transitional year. Many other elective transitional years, offering multidisciplinary perspectives on a certain theme, are conceivable. Similar to the ACTY, these could attract students motivated to develop a specific profile of competence while exploring career options within an area of medicine.

Elective thematic final years present a promising way to design the transition phase from student to doctor. This phase has the potential for professional transformation if learning opportunities are fully exploited [5, 31]. To seize opportunities, this phase requires a comprehensive curriculum of experiential learning and articulated goals [31–33]. Students will get opportunities to pursue personal interests, explore specialty preferences, and gain confidence in graduated responsibilities in patient care [31]. And, this final phase will offer opportunities to promote optimal preparation for clinical practice [5, 26, 31–33].

The ACTY meets many of the requirements of a transformative final phase. Still, awarding graduated responsibilities in acute care tasks is limited by legal and practical restrictions. Student training must not endanger the critically ill patient or delay needed treatment.

Growing into bearing acute care responsibility starts with experiential learning in a supervised setting. However, hands-on learning opportunities in time-pressured and infrequent clinical situations are scarce. Lack of opportunities poses a pivotal problem in undergraduate acute care training, with final year medical students reporting one to two hands-on experiences in providing acute care [34, 35]. Even in ACTY, we found that students have limited opportunities for frontline acute care experiences [24]. Affording acute care experiential learning, with progressive responsibilities in a supervised setting, may improve ACTY’s preparatory efficacy. The ACTY EPAs would have been suitable tools to apply progressive responsibilities in workplace learning.

However, the introduction of the ACTY and ACTY EPAs in 2015 preceded the adoption of the core undergraduate EPAs by UMC Utrecht medical school. The unfamiliarity of clinical supervisors with EPAs at the time may have impeded the exploitation of ACTY EPAs [24].

EPAs are a way to make accumulating clinical experience and competence visible from medical school to postgraduate training, including the ‘intercurricular’ period of working as a PNIT. Demonstrable competence could be taken into account in the individualization of postgraduate training (PGT) learning plans and could result in shortening of PGT time [15].

Since the implementation, the ACTY curriculum has been essentially unchanged. The ACTY design inspired the development of more thematic final years locally. This study and related research [24] call for a prominent role of the EPAs in ACTY workplace learning.

### Limitations

As part of a program evaluation, the quasi-experimental assessment data provide insight in graduates’ acute care performance. The study was not a controlled trial to prove the superiority of the intervention over the traditional curriculum. We did not control for confounding variables. For example, a study showed that students and interns who had taken an intensive care clinical elective performed better in a simulated test of acute care competence [28]. However, we applied strict inclusion criteria for all groups and measured pre-intervention performance, to constitute relevant comparisons.

Group sizes were relatively small. We had particular difficulty in recruiting comparison group students in the pre- and post-test set-up and reverted to including students for just the post-test, creating a larger but mixed control group. Student controls who did both pre- and post-tests did not score differently in the post-test compared to group ACTY, but this subgroup analysis lacks the power to conclude the absence of differences.

Depending on clinicians volunteering as assessors, we were unable to deploy multiple raters per station, rendering it impossible interrater reliabilities. Multiple raters per station might have reduced any bias and improved test reliability. However, different raters evaluated participants at different stations, so we assume the influence of rater bias on overall test results to be small. Although we extensively briefed each examiner prior to the assessment, we did not use an examiner team calibration training to prepare assessors.

The three-point global rating scale had the levels “does not meet expectations – borderline acceptable – meets expectations” (that faculty have of a PNIT with six months of clinical experience). An extra level for performance that “exceeded expectations” could have provided more discrimination between participants.

Lastly, the assessment was labor-intensive and costly, limiting sustainability and transferability.

This assessment study provides data on graduates’ acute care performance. In an ongoing study, we are surveying graduates’ perceptions of preparedness, on specialty orientation, and on the potential of the ACTY to shift career trajectories (i.e. shorter periods of working as a PNIT, shortening of residency training).

## Conclusion

In pursuit of a better transition to postgraduate expectations, we reported on a final year of medical school dedicated to acute care and found that graduates from this program show better resemblance to junior doctors with clinical experience than other graduates.

This final year dedicated to acute care enhances learning and preparedness for challenges that junior doctors in this area will face.

## Supplementary Information

**Additional file 1.**

**Additional file 2.**

**Additional file 3.**

**Additional file 4.**

**Additional file 5.**

**Additional file 6.**

**Additional file 7.**

## Data Availability

The datasets supporting the conclusions of this article are included within the article (and its additional files).

## Abbreviations

ACTY: Acute Care Transitional Year
CBD: Case-Based Discussion
ECG: Electrocardiogram
ED: Emergency Department
EPA: Entrustable Professional Activity
GRS: Global Rating Score
OSCE: Objective Structured Clinical Examination
PGT: PostGraduate Training
PNIT: Physician Not-In-Training
