# Supplementary material for: In pursuit of a better transition to selected residencies: a quasi-experimental evaluation of a final year of medical school dedicated to the acute care domain
Source: BMC Med Educ. 2022 Nov 23;22:807. doi: 10.1186/s12909-022-03871-0 (PMC9684806; doi:10.1186/s12909-022-03871-0)
Supplement: Supplementary file 1 — Additional file 1. [file 12909_2022_3871_MOESM1_ESM.docx]

**Appendix 1**

**Detailed description of ACTY EPAs**

| **Title** | Recognition and initial treatment of patients with  vital instability |
| --- | --- |
| **Description** | - Evaluate patient with the ‘ABCD’ approach; - Measure and interpret vital signs; - Based on the above, come to a clinical evaluation; - In case of emergency: call directly for help/supervision; - Initiate Basic Life Support, perform bag-mask-ventilation and airway maneuvers and start intravenous fluid therapy; - Be a member of the resuscitation team; - Discern limitations in capability.   This EPA does not comprise:   - Provision of advanced cardiac/trauma life support, completely and/or indirectly supervised - Care for pediatric patients with vital instability (< 16 years) |
| **Link to competency domains** | CanMEDS (Dutch KNMG version):   - Medical Expert: applies diagnostic, therapeutic, preventive repertoire (1.2) - Communicator: reports adequately on a case, orally and in writing (2.4) - Collaborator: consults others effectively (3.1), efficacious in interdisciplinary collaboration (3.4) - Manager: works effectively within a system (6.2) - Professional: discerns limits of competence (7.3) |
| **Required knowledge, skills, and attitudes (level of doctor in postgraduate year 1)** | *Knowledge*   - Knowledge of normal ranges for blood pressure, heart rate, respiratory rate, saturation and temperature; - Knowledge of symptoms of threatened vital parameters - Knowledge of Glasgow Coma Scale (GCS) and primary neurological survey; - Knowledge of causes of disturbances in consciousness; - Knowledge of Basic Life Support; - Knowledge of principles of advanced cardiac and trauma life support; - Knowledge of 4Hs, 4Ts, shock and no-shock algorithms; - Knowledge of indications for and application of (Automatic) External Defibrillators; - Knowledge of effective communication protocols to call for help (e.g. SBARR method); - Knowledge of local protocols regarding acute intervention teams, emergency telephone number, resuscitation team etcetera; - Knowledge of equipment and materials for basic interventions in ‘ABC’ (e.g. infusion materials, AMBU-bag, non-rebreathing mask, Mayo tube) including (contra-)indications; - Knowledge of levels of care on different wards (normal ward to ICU); - Knowledge of indications to stop resuscitation efforts     *Skills*   - Asks timely for help/supervision with an effective communication protocol (e.g. SBARR) and locally applicable emergency procedure; - Performs and interprets a systematic physical exam according to ‘ABCD’ and monitoring of vital signs; - Clinical judgment based on ‘ABCD’ and vital parameters; - Provides probable diagnosis regarding the cause of the vital instability; - Gives and receives orders/tasks and confirms the order/task and its execution; - Proposes a management plan; - Proposes orders regarding treatment; - Initiates Basic Life Support; - Performs Basic Life Support skills and part-tasks; - Performs bag-mask ventilation and airway maneuvers (head tilt/chin lift, jaw thrust); - Sites a peripheral intravenous catheter; - Defibrillates a shockable rhythm with (automatic) external defibrillator; - Reports in medical record; - Provides a structured handover of a patient with vital instability to colleague/supervisor/ward.   *Attitudes*   - Discerns and acknowledges personal limits of knowledge, skill and capability and can adequately reflect on this; - Situational awareness; - Is set to short cycles of assessment, treatment, and re-assessment; - Collaborates in a team in an emergency setting; - Reveals professional role and level; - Professional conduct towards patient and/or relatives; - Uses Evidence Based Medicine. |
| **Information to assess progress** | *Workplace assessment*   - Mini-CEXs with regard to the evaluation of patients with or without vital instability, including indicating necessity for (acute) intervention and with regard to discernment of personal limits of capability; - Multisource feedback: performance as a team member in urgent and non-urgent settings;   *Assessment of knowledge, skills and attitudes*   - Knowledge examination (written, variety of formats); - Clinical reasoning and know-how (case-based discussions); - Demonstration of isolated skills in non-clinical setting (Objective Structured Examination of Clinical Skills); - Reflection forms regarding performance, difficult moments, discernment of limitations.   *Assessment of clinical performance*   - Simulation of acute care settings. |
| **Target level of supervision (entrustment)** | Indirect supervision (immediately available): evaluation and initial management, awaiting arrival of help/supervisor |
| **When is unsupervised practice expected?** | At the end of DTY Acute Care |

| **Title** | Evaluation of patients with respiratory insufficiency |
| --- | --- |
| **Description** | - Rapid evaluation with the ‘ABCD’ approach; - Take a focused history of a patient with respiratory insufficiency in an Emergency Room, Coronary Care Unit, ward or urgent outpatient clinic (outside Operating Room and Intensive Care settings) ; - Perform a physical exam, including ‘ABCD’ and vital signs; - Order and interpret *basic* diagnostic tests (blood, chest X-ray, ECG); - Draft and rank a differential diagnosis in a patient with respiratory insufficiency; - Propose initial management and treatment plans.   This EPA does not comprise:   - Care for the patient (nearly) in a cardiopulmonary resuscitation setting - Care for pediatric patients with respiratory instability (< 16 years) - Interpretation of advanced diagnostic tests (Ventilation-Perfusion scans, spirometry) |
| **Link to competency domains** | CanMEDS (Dutch KNMG version):   - Medical Expert: applies diagnostic, therapeutic, preventive repertoire (1.2) - Communicator: creates effective therapeutic relationships (2.1), reports adequately on a case, orally and in writing (2.4) - Collaborator: consults others effectively (3.1) - Scholar: appraises medical information critically (4.1) - Health Advocate: recognizes determinants of disease (5.1) - Manager: works effectively within a system (6.2) - Professional: adequate (inter-)professional conduct (7.2), discerns limits of competence (7.3) |
| **Required knowledge, skills, and attitudes (level of doctor in postgraduate year 1)** | *Knowledge*   - Knowledge of normal ranges for blood pressure, heart rate, respiratory rate, saturation and temperature; - Knowledge of relevant criteria and scores (e.g. SIRS-criteria, Wells score; GOLD-criteria; Pneumonia Severity Index; Pneumonia Likelihood Ratio; AMBU-65 and CURB-65; ALI/ARDS); - Knowledge of criteria for ventilation and admission to ward or ICU; - Knowledge of (results of) relevant diagnostic tests (i.e. laboratory blood tests, blood gas analysis, chest X-ray and systematic interpretation, ECG); - Knowledge of results of pleural fluid investigations (chemistry, microbiology); - Knowledge of most important disorders leading to respiratory insufficiency; - Knowledge of options in additional diagnostic testing (e.g. rapid PCR tests, viral, bacterial and fungal tests, CT scan, VP scan); - Knowledge of indications for ventilation; - Knowledge of indications and techniques of oxygen therapy (F_i_O_2_, nasal cannulas, non-rebreathing mask, CPAP, Venturi, Optiflow); - Knowledge of indications for chest drains; - Knowledge of indications for bronchoscopy; - Knowledge of initial pharmacological treatment of important disorders leading to respiratory insufficiency.   *Skills*   - Performs and interprets a systematic physical exam according to ‘ABCD’ and monitoring of vital signs; - Asks timely for help/supervision; - Takes a focused history and performs a focused physical exam in patients with respiratory insufficiency; - Performs a blood gas analysis; - Interprets result of a blood gas analysis; - Connects patient to monitoring such as ECG/telemetry, non-invasive blood pressure, pulse oximetry, and interprets findings; - Interprets 12-lead ECG: myocardial ischemia, rhythm- and conduction disorders; - Recognizes myocardial ischemia, rhythm- and conduction disorders on telemetry monitor; - Writes an order for chest X-ray; - Systematic interpretation of chest X-ray and recognition of relevant radiologic findings; - Interprets findings of pleural fluid tests; - Sites a peripheral intravenous catheter; - Drafts and ranks a differential diagnosis and provides a probable diagnosis in a patient with circulatory insufficiency; - Proposes a management plan; - Proposes orders regarding treatment; - Reports in medical record and provides a structured handover.   *Attitudes*   - Discerns and acknowledges personal limits of knowledge, skill and capability and can adequately reflect on this; - Is set to short cycles of assessment, treatment, and re-assessment; - Reveals professional role and level; - Collaborates with ward staff; - Professional conduct towards patient and/or relatives; - Uses Evidence Based Medicine. |
| **Information to assess progress** | *Workplace assessment*   - Mini-CEXs with regard to the evaluation of patients with or without vital instability, including indicating necessity for (acute) intervention and with regard to discernment of personal limits of capability; - Multisource feedback: performance as a team member in urgent and non-urgent settings;   *Assessment of knowledge, skills and attitudes*   - Knowledge examination (written, variety of formats); - Clinical reasoning and know-how (case-based discussions); - Demonstration of isolated skills in non-clinical setting (Objective structured examination of skills); - Reflection forms regarding performance, difficult moments, discernment of limitations.   *Assessment of clinical performance*   - Simulation of acute care settings. |
| **Target level of supervision (entrustment)** | Indirect supervision (immediately available): evaluation and initial management, awaiting arrival of help/supervisor |
| **When is unsupervised practice expected?** | At the end of DTY Acute Care |

| **Title** | Evaluation of patients with circulatory insufficiency |
| --- | --- |
| **Description** | - Rapid evaluation with the ‘ABCD’ approach; - Take a focused history of a patient with circulatory insufficiency in an Emergency Room, Coronary Care Unit, ward or urgent outpatient clinic (outside Operating Room and Intensive Care settings) ; - Perform a physical exam, including ‘ABCD’ and vital signs; - Order and interpret *basic* diagnostic tests (blood, chest X-ray, ECG); - Draft and rank a differential diagnosis in a patient with circulatory insufficiency; - Propose initial management and treatment plans.   This EPA does not comprise:   - Care for the patient (nearly) in a cardiopulmonary resuscitation setting - Care for pediatric patients with circulatory instability (< 16 years) - Interpretation of advanced diagnostic tests (cardiac catheterization, echocardiography) |
| **Link to competency domains** | CanMEDS (Dutch KNMG version):   - Medical Expert: applies diagnostic, therapeutic, preventive repertoire (1.2) - Communicator: creates effective therapeutic relationships (2.1), reports adequately on a case, orally and in writing (2.4) - Collaborator: consults others effectively (3.1) - Scholar: appraises medical information critically (4.1) - Health Advocate: recognizes determinants of disease (5.1) - Manager: works effectively within a system (6.2) - Professional: adequate (inter-)professional conduct (7.2), discerns limits of competence (7.3) |
| **Required knowledge, skills, and attitudes (level of doctor in postgraduate year 1)** | *Knowledge*   - Knowledge of normal ranges for blood pressure, heart rate, respiratory rate, saturation and temperature; - Knowledge of relevant criteria and scores (e.g. Crusade, Heartscore, TIMI, CHADS2VASC, SIRS-criteria, hypovolemic shock classification, NYHA classification); - Knowledge of different types of shock and their treatments; - Knowledge of levels of care on different wards (normal ward to ICU); - Knowledge of (results of) relevant diagnostic tests (i.e. laboratory blood tests, blood gas analysis, chest X-ray and systematic interpretation, ECG); - Knowledge of intravenous fluid therapy protocols; - Knowledge of most important disorders leading to circulatory insufficiency (i.e. different types of shock); - Knowledge of indications for echocardiography and of relevant reported findings; - Knowledge of indications for cardiac catheterization (and percutaneous cardiac intervention) and of relevant reported findings; - Knowledge of initial (pharmacological) treatment of different types of shock. Inotropes and vasoactive medications.   *Skills*   - Performs and interprets a systematic physical exam according to ‘ABCD’ and monitoring of vital signs; - Asks timely for help/supervision; - Takes a focused history and performs a focused physical exam in patients with circulatory insufficiency; - Performs a blood gas analysis; - Interprets result of a blood gas analysis; - Connects patient to monitoring such as ECG/telemetry, non-invasive blood pressure, pulse oximetry, and interprets findings; - Evaluates heart rhythm on telemetry monitor; - Interprets 12-lead ECG : myocardial ischemia, rhythm- and conduction disorders; - Writes an order for chest X-ray; - Systematic interpretation of chest X-ray and recognition of relevant radiologic findings; - Sites a peripheral intravenous catheter; - Drafts and ranks a differential diagnosis and provides a probable diagnosis in a patient with circulatory insufficiency; - Proposes a management plan; - Proposes orders regarding treatment; - Reports in medical record and provides a structured handover.   *Attitudes*   - Discerns and acknowledges personal limits of knowledge, skill and capability and can adequately reflect on this; - Is set to short cycles of assessment, treatment, and re-assessment; - Reveals professional role and level; - Collaborates with ward staff; - Professional conduct towards patient and/or relatives; - Uses Evidence Based Medicine. |
| **Information to assess progress** | *Workplace assessment*   - Mini-CEXs with regard to the evaluation of patients with or without vital instability, including indicating necessity for (acute) intervention and with regard to discernment of personal limits of capability; - Multisource feedback: performance as a team member in urgent and non-urgent settings;   *Assessment of knowledge, skills and attitudes*   - Knowledge examination (written, variety of formats); - Clinical reasoning and know-how (case-based discussions); - Demonstration of isolated skills in non-clinical setting (Objective Structured Examination of Clinical Skills); - Reflection forms regarding performance, difficult moments, discernment of limitations.   *Assessment of clinical performance*   - Simulation of acute care settings. |
| **Target level of supervision (entrustment)** | Indirect supervision (immediately available): evaluation and initial management, awaiting arrival of help/supervisor |
| **When is unsupervised practice expected?** | At the end of DTY Acute Care |
