# Supplementary material for: In pursuit of a better transition to selected residencies: a quasi-experimental evaluation of a final year of medical school dedicated to the acute care domain
Source: BMC Med Educ. 2022 Nov 23;22:807. doi: 10.1186/s12909-022-03871-0 (PMC9684806; doi:10.1186/s12909-022-03871-0)
Supplement: Supplementary file 2 — Additional file 2. [file 12909_2022_3871_MOESM2_ESM.docx]

**Appendix 2**

**Detailed description of the multimodal acute care assessment**

Participants took part in a multimodal acute care assessment in which they were evaluated at junior doctor-level, i.e. the expected level of a recent graduate with six months clinical experience.

Specifically for ACTY and this study, expert clinicians of the five specialties participating in ACTY collaboratively designed a multimodal assessment inspired by the levels of Miller’s pyramid: Knows, Knows how, Shows how, and Does [1]. We chose to assess these components of competence as a proxy for assessing competence. Because we required highly comparable assessments of all participants under controlled circumstances, we were not able to use clinical workplace assessments to evaluate participants at the Does level. Instead, we used high fidelity simulations in contextually rich scenarios of acutely compromised patients to mimic clinical practice. Simulations can be considered to evaluate performance high in the Shows how level, approaching the Does level[2].

The clinical learning objectives of the Acute Care program, formulated as Entrustable Professional Activities (EPAs) and describing authentic acute care tasks that junior doctors face, served as assessment blueprint. The EPAs listed the knowledge, skills, and attitudes required for execution of the task [3]. Each listed aspect formed a row in the blueprint matrix and linked to one or more assessment elements, forming the columns of the matrix.

We assessed Knows level with a knowledge test, Knows how level with case-base discussions (CBDs), Shows how with skills stations in Objective Structured Clinical Examination format, and Higher shows how level with high-fidelity simulations.

***Assessment modes***

*Knowledge test*

This was a 40 minute paper-based written test, containing around 40 closed and six open format questions, targeting factual or applied knowledge or higher order thinking. Items requiring application or higher order thinking yielded more points than factual items. We created new questions or drew upon ACTY faculty databases, and one of the authors (GJ) and an assessment expert evaluated and amended items as needed. Three versions of parallel content existed.

*CBDs*

Through one-on-one structured questioning and discussion, assessors evaluated the process of information gathering, clinical reasoning and know-how of case management. ACTY faculty devised four cases describing patients requiring urgent attendance at the Emergency Department. Faculty and GJ fine-tuned cases iteratively. Each case had a specific detailed checklist following the chronology of the consultation, with anchors describing essential steps or expected actions. Examiners scored the performance on a three-point scale (done adequately, done incompletely or not timed well, done insufficiently or omitted). Specific aspects, such as appropriate prioritization, completeness, or clinical overview, could be awarded with extra points, where indicated on the checklist. Assessors completed the paper-based scoring checklist during and directly after the discussion.

Participants did two CBDs of ten minutes duration, with two different assessors, on patients requiring urgent attendance at the Emergency Department. We used two sets of two cases.

*OSCEs*

This part of the assessment consisted of five to seven skills stations of five minutes duration. ACTY faculty devised several stations in OSCE format for skills mentioned in the learning objectives. Each station had a specific detailed checklist made up of the consecutive steps in the execution of the skill. Assessors rated performance on a three-point scale (done adequately, done incompletely or not timed well, done insufficiently or omitted. Assessors completed the paper-based scoring checklist during and directly after skill performance.

*Simulations*

The part, resembling clinical work most, consisted of three real-time simulation scenarios, each lasting approximately 12 minutes, run by anesthesiologists and anesthetic nurses who were all EUSim or CMS certified simulation facilitators ([www.eusim.org](file:///\\DS\HOME\VF\gjonker4\Home\EPA\LIC\Pre-post-test\www.eusim.org) and www.harvardmedsim.org). Anesthesiologists from the simulation center developed scenarios in collaboration with ACTY faculty and one of the authors (GJ). Scenarios used a standardized script and were enacted to occur in the emergency room or ward and were tasks that a physician-not-in-training could encounter. The participant enacted the role of the first responding doctor and a nurse was present who acted only on instruction.

The simulator room mimicked a room in a clinical environment and deployed a 2010 Laerdal Sim Man (Laerdal Benelux B.V., Amersfoort, the Netherlands). The high-fidelity manikin featured a wide range of sounds and pulsations and could be connected to a monitor. Props such as white coats, drug carts, telephone system, disposables and relevant working equipment resulted in an authentic hospital-like environment [4].

We devised detailed scenario-specific checklists of expected actions and important steps in the management of the case. Assessment, by the simulation center faculty, entailed clinical reasoning, clinical judgment and technical and behavioral skills including communication and crisis resource management. We did not use video recordings. Assessors completed the paper-based scoring checklist during and directly after skill performance.

Facilitators debriefed participants after each scenario to foster learning and well-being.

***Pre- and post-test***

Every six weeks, students could commence in their transitional year and, normally, would graduate a year later. Therefore, we organized a test instance every six weeks. At one instance, all participants took the same test, either as pre-test or post-test. Participants in the intervention group and student control group attended the pre-test before starting in their final year rotations and the post-test in about the last month of their final year. We informed all participants about the aim of the study and assessment procedures. Participants agreed not to disclose exam content to their peers.

To complete the assessment participants attended two separate half-days within a timeframe of one week. On the first half-day, the knowledge test, CBDs and OSCEs took place at University Medical Center Utrecht, the Netherlands. With preparation and rest stations, this part had a duration of three hours. On the second half-day, simulations took place at the simulation center of Rijnstate Hospital, Velp, the Netherlands. With induction, briefing, rest and waiting time and debriefing, this part took about three hours.

One of the authors (GJ) supervised all assessment instances, assuring that the assessment elements together were a representative coverage of the domain. We ran 25 test instances, which were highly similar. Participants’ pre-test content was different from their post-test. Marshalls coordinated the assessment. They managed time protocols to create equal opportunities for participants between instances and reduce random and systematic error.

Within the ACTY, the pre- and post-test were formative and results had no formal consequences for students. Purpose of the pre-test, from a curricular point of view, was to identify personal performance gaps, force focus on the learning objectives, and confront students with the expected performance level of junior doctors. Therefore, all participants received feedback on their performance in the tests.

*Examiners*

Assessors at CBDs and OSCEs were all clinicians – trainees or specialists – with appropriate expertise and experience for the stations at which they examined. They volunteered when available. The investigators briefed all examiners individually on the use of the scoring rubric, expected performance level and station duration. Examiners were not made aware whether participants were from intervention or control groups, nor whether participants did a pre- or post-test. Examiners scored participants’ performance live on a paper checklist.

***Appendix references***

1. Miller GE. The assessment of clinical skills/competence/performance. Acad Med. 1990;65(9):S63-S7.

2. Ten Cate O, Carraccio C, Damodaran A, Gofton W, Hamstra SJ, Hart D, et al. Entrustment Decision Making: Extending Miller's Pyramid. Acad Med. 2020.

3. Ten Cate O. Nuts and bolts of entrustable professional activities. J Grad Med Educ. 2013;5(1):157-8.

4. Groot F, Jonker G, Rinia M, Ten Cate O, Hoff RG. Simulation at the Frontier of the Zone of Proximal Development: A Test in Acute Care for Inexperienced Learners. Acad Med. 2020.
