# Supplementary material for: In pursuit of a better transition to selected residencies: a quasi-experimental evaluation of a final year of medical school dedicated to the acute care domain
Source: BMC Med Educ. 2022 Nov 23;22:807. doi: 10.1186/s12909-022-03871-0 (PMC9684806; doi:10.1186/s12909-022-03871-0)
Supplement: Supplementary file 3 — Additional file 3. [file 12909_2022_3871_MOESM3_ESM.docx]

**Appendix 3**

**Detailed report of statistical analyses**

Pre-test, post-test and gain in mean scores on knowledge, CBDs, OSCEs, and simulations (in percentage of maximum score).

|  | **Group ACTY** | | | | | | **Group C** | | | | | | **Group P** | |
| --- | --- | --- | --- | --- | --- | --- | --- | --- | --- | --- | --- | --- | --- | --- |
|  | **Pre** | | **Post** | | **Gain** | | **Pre** | | **Post** | | **Gain** | | **Post** | |
| **Assessment modality** | **n** | **Mean (SD)**  **95% CI** | **n** | **Mean (SD)**  **95% CI** | **n** | **Mean (SD)**  **95% CI** | **n** | **Mean (SD)**  **95% CI** | **n** | **Mean (SD)**  **95% CI** | **n** | **Mean (SD)**  **95% CI** | **n** | **Mean (SD)**  **95% CI** |
| Knowledge | 33 | 59 (8)  56 – 62 | 29 | 67 (11)  63 – 71 | 29 | 8 (14)  3 – 14 | 10 | 53 (8)  47 – 59 | 19 | 63 (13)  56 – 69 | 8 | 11 (14)  -1 – 23 | 16 | 67 (7)  63 – 70 |
| CBDs | 33 | 64 (12)  60 – 68 | 29 | 79 (11)  75 – 84 | 29 | 16 (11)  12 – 20 | 10 | 62 (12)  53 – 71 | 19 | 72 (10)  67 – 77 | 8 | 13 (14)  1 – 24 | 16 | 79 (9)  74 – 84 |
| OSCEs | 33 | 64 (7)  61 – 67 | 29 | 74 (9) ***** 71 – 78 | 29 | 10 (9)  6 – 14 | 10 | 62 (6)  57 – 66 | 19 | 67 (8) ******†* 63 – 70 | 8 | 10 (5)  6 – 14 | 16 | 72 (5) *†* 69 – 75 |
| Simulations | 33 | 51 (6) *††* 49 – 53 | 27 | 65 (8) ‡ ******  62 – 69 | 27 | 14 (11)  9 – 18 | 10 | 46 (8) *††* 40 – 51 | 14 | 59 (11) ‡ **§** 52 – 65 | 6 | 13 (17)  -6 – 31 | 17 | 71 (6) **§ ****  68 – 74 |

*ACTY= Acute Transitional Year group, C= student comparison group, P= PNIT comparison group. Pre= pre-test, Post= post-test. Gain= difference between post-test and pre-test. Assessment modality scores expressed as % of maximum obtainable score, as group mean (SD= standard deviation) and 95% confidence interval (CI). PNITs did not participate in the pre-test, for comparison with groups ACTY and C we label their participation as post-test. The focus in the paper is on post-test comparison. ** *p=.004; †* *p=.026;* ‡ *p=.036;* § p<.001; *** p=.019; †† p=.026*

**Pre-test**

Only group ACTY and (part of) group C took part in the pre-test.

There were no statistically significant differences in group means on knowledge, CBDs, and OSCEs, as determined by one-way ANOVA. There was a statistically significant difference between groups on simulations, as determined by one-way ANOVA.

| **Assessment modality** | **ANOVA F(1,41)** | **p** |
| --- | --- | --- |
| Knowledge | 3.822 | .057 |
| CBDs | .221 | .641 |
| OSCEs | .957 | .334 |
| Simulations | 5.355 | .026 |

Group ACTY had statistically significant higher group mean on simulations than group C, as determined by independent samples t-test: t(41)=2.314, p=.026, mean difference 6 (2), 95% CI 1 – 11.

**Post-test**

On knowledge and CBDs, there were no statistically significant differences between groups, as determined by one-way ANOVA. However, on OSCEs and simulations, there were statistically significant differences between groups, as determined by one-way ANOVA.

| **Assessment modality** | **ANOVA** | **p** |
| --- | --- | --- |
| Knowledge | F(2,61)=0.941 | .396 |
| CBDs | F(2,61)=3.272 | .05 |
| OSCEs | F(2,61)=5.587 | .01 |
| Simulations | F(2,55)=8.239 | .001 |

On OSCEs, group ACTY had statistically significant higher group mean than group C, as determined by independent samples t-test: t(46)=3.068, p=.004, mean difference 8 (3), 95% CI 3 – 13.

Group P had statistically significant higher group mean on OSCEs than group C, as determined by independent samples t-test: t(33)=2.325, p=.026, mean difference 5 (2), 95% CI 1 – 10.

On simulations, group ACTY had statistically significant higher group mean than group C, as determined by independent samples t-test: t(39)=2.172, p=.036, mean difference 7 (3), 95% CI 1 – 13.

Group P had statistically significant higher group mean than group C, as determined by independent samples t-test: t(29)=4.089, p<.001, mean difference 12 (3), 95% CI 6 – 18.

Group P also had statistically significant higher group mean than group ACTY, as determined by independent samples t-test: t(42)=2.430, p=.019, mean difference 6 (2), 95% CI 1 – 10.

**Gain**

In gain between pre-test and post-test scores, there was no statistically significant difference between means of group ACTY and group C on all assessment modalities, as determined by one-way ANOVA.

| **Assessment modality** | **ANOVA** | **p** |
| --- | --- | --- |
| Knowledge | F(1,35)=0.287 | .596 |
| CBDs | F(1,35)=0.514 | .478 |
| OSCEs | F(1,35)=0.002 | .964 |
| Simulations | F(1,31)=0.035 | .853 |

**Post-test GRS**

In addition to checklist scores, on CBDs, OSCEs and simulations, assessors evaluated participant performance on a three-point global rating score (does not meet expectations – borderline - meets expectations), in answer to the question “How did the participant’s performance compare to the expected level of a PNIT with six months of clinical experience?”.

We used a cumulative odds ordinal logistic regression with proportional odds to determine the effect of group type on receiving a high GRS.

The deviance goodness-of-fit test indicated that the model was a good fit to the observed data of CBDs, χ^2^(2)= 1.37, p=.504, OSCEs, χ^2^ (2)= 1.01, p=.603, and simulations, χ^2^ (2)=.621, p=.733. The final model statistically significant predicted the dependent variable over and above the intercept-only model for CBDs χ^2^ 7.37, p=.023, OSCEs χ^2^ 12.24, p=.002, and simulations χ^2^ 21.62, p<.001.

Group type had a statistically significant effect on the prediction of getting a high GRS on CBDs, Wald χ^2^ (2)=6.69, p=.035, on OSCEs, Wald χ^2^ (2)=12.44, p=.002, and on simulations, Wald χ^2^ (2)=19.43, p<.001.

Compared to group C, the OR of participants in group ACTY getting a high GRS was 2.49 for CBDs, 2.47 for OSCEs, and 2.06 for simulations; statistically significant for OSCEs and simulations. The OR of PNITs to get a high GRS, compared to group C, was 6.69 for CBDs, 2.45 for OSCEs, and 6.57 for simulations; all statistically significant.

|  | **Group ACTY** | | | **Group C** | | | **Group P** | | |
| --- | --- | --- | --- | --- | --- | --- | --- | --- | --- |
| **Assessment modality** | **OR** | **95% CI** | **Wald χ^2^ (1)** | **OR** | **95% CI** | **Wald χ^2^ (1)** | **OR** | **95% CI** | **Wald χ^2^ (1)** |
|  |  |  | **p** |  |  | **p** |  |  | **p** |
| CBDs | 2.49 | .92 – 6.72 | 3.21 | 1.0 | ref | - | 6.69 | 1.35 – 33.21 | 5.40 |
|  |  |  | .073 |  |  | - |  |  | .020 |
| OSCEs | 2.47 | 1.44 – 4.25 | 10.66 | 1.0 | ref | - | 2.45 | 1.28 – 4.71 | 7.23 |
|  |  |  | .001 |  |  | - |  |  | .007 |
| Simulations | 2.06 | 1.03 – 4.14 | 4.14 | 1.0 | ref | - | 6.57 | 2.83 – 15.25 | 19.21 |
|  |  |  | .042 |  |  | - |  |  | <.001 |

*Group C is reference category with a set OR of 1.0.*

The odds of PNITs getting a high GRS on simulations was 3.22 times that of DTY students, a statistically significant effect. For CBDs and skills, the odds did not differ significantly from DTY students (OR 0.37 and OR 1.01, respectively).

|  | **Group ACTY** | | | **Group P** | | |
| --- | --- | --- | --- | --- | --- | --- |
| **Assessment modality** | **OR** | **95% CI** | **Wald χ^2^ (1)** | **OR** | **95% CI** | **Wald χ^2^ (1)** |
|  |  |  | **p** |  |  | **p** |
| CBDs | 1.0 | ref | - | 2.70 | 0.54 – 14.29 | 1.46 |
|  |  |  | - |  |  | .228 |
| OSCEs | 1.0 | ref | - | 0.99 | 0.53 – 1.85 | .001 |
|  |  |  | - |  |  | .979 |
| Simulations | 1.0 | ref | - | 3.22 | 1.52 – 6.67 | 9.48 |
|  |  |  | - |  |  | . 002 |

*Group ACTY is reference category with a set OR of 1.0. Group C left-out as comparison ACTY vs C was made above.*

In summary, both ACTY graduates and PNITs had statistically significant higher odds to get a high GRS than graduates form group C on skills and simulations. PNITs had statistically significant higher odds to get a higher GRS on CBDs, as well. PNITs also had statistically significant higher odds to get a high GRS on simulations than ACTY graduates.
