# Supplementary material for: In pursuit of a better transition to selected residencies: a quasi-experimental evaluation of a final year of medical school dedicated to the acute care domain
Source: BMC Med Educ. 2022 Nov 23;22:807. doi: 10.1186/s12909-022-03871-0 (PMC9684806; doi:10.1186/s12909-022-03871-0)
Supplement: Supplementary file 4 — Additional file 4. [file 12909_2022_3871_MOESM4_ESM.docx]

**Legend to Inpursuitofabettertransition_rotations**

**Case:**

ID

**Type:**

1= ACTY, 2= non-ACTY controls, 3= PNITs

**Sex:**

1=female, 2=male, 3=not-specified

**12 week clinical rotation, 6 week clinical electives, 12 week research rotation, 6 week extracurricular block, other block or PNIT Job in:** 1=anesthesiology, 2=cardiology, 3=emergency medicine, 4=intensive care medicine, 5=pulmonology, 6=surgery, 7=pediatrics, 8=internal medicine, 9=microbiology, 10=plastics, 11=ophthalmology, 12=neurology, 13=geriatrics, 14=general practice, 15=sports medicine, 16=medical education, 17=psychiatry, 999= not-specified

**Months:**

Number of months in PNIT job
