# Supplementary material for: In pursuit of a better transition to selected residencies: a quasi-experimental evaluation of a final year of medical school dedicated to the acute care domain
Source: BMC Med Educ. 2022 Nov 23;22:807. doi: 10.1186/s12909-022-03871-0 (PMC9684806; doi:10.1186/s12909-022-03871-0)
Supplement: Supplementary file 5 — Additional file 5. [file 12909_2022_3871_MOESM5_ESM.docx]

**Legend to Inpursuitofabettertransition_datasetfull**

***Variables in order of appearance***

**Case:**

ID

**Type:**

1= ACTY, 2= non-ACTY controls, 3= PNITs

**Sex:**

1=female, 2=male, 3=not-specified

***Knowledge test***

Knowledge (1) pre-test (.1) test item version (.1,2 or 3)

e.g. Score.1.1.1, Score.1.1.2, Score.1.1.3

**Knowledge pre-test summary score**

ScoreKpre

Knowledge (1) post-test (.2) test item version (.1,2 or 3)

e.g. Score.1.2.1

**Knowledge post-test summary score**

ScoreKpost

**Difference between post- and pre- knowledge summary scores**

deltaK

***Case-based discussions***

Percentage score on CBD (2) pretest (.1) test item version (.4, 5, 6 or 7)

e.g. Score 2.1.4

GRS on CBD (2) pretest (.1) version (.4, 5, 6 or 7)

1=fail, 2=borderline, 3=pass

**CBD pre-test summary score**

preCBDscore

Percentage score on CBD (2) posttest (.2) test item version (.4, 5, 6 or 7)

GRS on CBD (2) posttest (.2) test item version (.4, 5, 6 or 7)

1=fail, 2=borderline, 3=pass

**CBD post-test summary score**

postCBDscore

**Difference between post- and CBD summary scores**

deltaCBD

***OSCEs***

Percentage score on OSCE (3) pretest (.1) test item version (.10 - .18)

e.g. Score3.1.10

GRS on OSCE (3) pretest (.1) version (.10 - .18)

1=fail, 2=borderline, 3=pass

Percentage score on OSCE (3) posttest (.1) test item version (.10 - .18)

GRS on OSCE (3) posttest (.2) test item version (.10 - .18)

1=fail, 2=borderline, 3=pass

***Simulations***

Percentage score on Simulations (4) pretest (.1) test item version (.19 - .27)

e.g. Score4.1.19

GRS on Simulations (4) pretest (.1) version (.19 - .27)

1=fail, 2=borderline, 3=pass

**Simulation pre-test summary score**

preSimscore

Percentage score on Simulations (4) posttest (.2) test item version (.19 - .27)

GRS on Simulations (4) posttest (.2) test item version (.19 - .27)

1=fail, 2=borderline, 3=pass

**Simulations post-test summary score**

postSimscore

**Difference between post- and pre simulation summary scores**

deltaSim

***OSCEs (cont.)***

**OSCE pre-test summary score**

preSscore

**OSCE post-test summary score**

postscore

**Difference between post- and pre OSCE summary scores**

deltaS
